# Exploring Multiple Barriers to Proper Child Feeding Practices in Rural Districts of Ethiopia

**DOI:** 10.1002/fsn3.4757

**Published:** 2025-02-25

**Authors:** Kassahun Fikadu, Manaye Yihune, Wanzahun Godana Boynito, Zeleke Hailemariam

**Affiliations:** ^1^ Department of Midwifery Arba Minch University Arba Minch Ethiopia; ^2^ School Public Health Arba Minch University Arba Minch Ethiopia

**Keywords:** barriers, child feeding practices, cultural, Gamo zone, socioeconomic

## Abstract

Infants' and young children's health and development rely on optimal feeding techniques. Malnutrition is the leading cause of preventable illness and death in infants and early childhood. This impact is mostly significant in low‐ and middle‐income countries, where childhood illness and mortality rates have risen considerably. To explore the barriers to child feeding practices in the rural Gamo zone, south Ethiopia, from primary caregivers and different key individuals' perspectives. A phenomenological qualitative study approach with a purposive sampling technique was carried out to explore the barriers and facilitators of child feeding practices in the study area from August 14 to September 10, 2023. Data was collected from fathers, health extension workers, the health development army, religious leaders, and community elders who reside in rural communities of the Gamo zone. Three focus group discussions, including 8–10 participants per group with females and three with males until data saturation. A total of 51 discussants and 12 key informants participated in the study. The following barriers were identified from the study. Limited income and employment opportunities, lack of property ownership, limited maternal and husband education, and lack of knowledge about proper child feeding practices are among the factors leading to inadequate nutrition for children. Large family sizes, marriage‐related factors like polygamy, early marriage, limited freedom of partner choice, and gender inequalities can affect necessary support for appropriate child feeding practices and negatively impact children's nutrition. Moreover, cultural norms, religious beliefs, lack of awareness regarding the husband's income, and lack of support from family members or communities were identified as barriers that influence child feeding practices. The study unveiled three key themes that impede the adoption of appropriate child feeding practices: economic status, demographic conditions, and sociocultural aspects that affect the feeding practices of children. Effective interventions to improve child feeding practices must consider and address these identified barriers.

## Graphical abstract

This study reveals how economic hardships, gender inequities, and cultural beliefs shape child feeding practices in rural Ethiopia. The findings call for collaborative efforts to overcome these challenges and promote healthier outcomes for children.

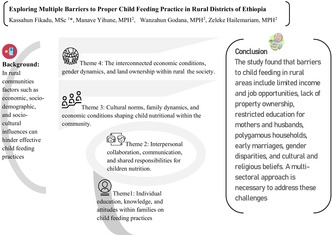

## 1 Introduction

Malnutrition and poor complementary feeding are more predominant in rural than urban areas. Major determinants of appropriate feeding practices in this study were the age of the index children, the child's birth order, and the mother's income (Ariyo et al. 2021). Childhood undernutrition remains a major public health problem in many developing countries, contributing to high prevalence of stunting, poor cognitive development, and increased morbidity (Black et al. 2008).

Child malnutrition remains a global health challenge though the burden and severity are higher and more pronounced in developing countries like Ethiopia, thereby resulting in high morbidity and mortality, and growth failure (Akombi et al. 2017; Organization and UNICEF 2003). Inappropriate feeding practices remain an impediment affecting a large number of children and thereby hindering their growth and development (Bégin and Aguayo 2017).

There is a growing agreement that good nutrition during the window period of the first 1000 days of life is critical to achieving full human potential throughout life, and a means to break the intergenerational cycle of poverty and inequity. Accordingly, the World Health Assembly (WHA) in 2012 endorsed a Comprehensive Implementation Plan on Maternal, Infant, and Young Child Nutrition with six global targets, which have also been incorporated in the post‐2015 sustainable development agenda (Asma et al. 2020; Sachs 2012). Despite this encouraging global momentum, recent estimates showed that current progress in reducing child undernutrition in many Low and Middle‐income countries (LMICs) is inadequate to achieve the WHA global targets (De Onis and Branca 2016).

According to the global burden of maternal and child undernutrition estimates in 2020, globally, 149.2 million children under the age of 5 years were stunted, 45.4 million were wasted (UNICEF 2019). Growth faltering in early life is associated with an elevated risk of mortality, poor neurocognitive development, poor learning capacity and productivity in later life, and increased risk of nutrition‐related chronic diseases during adulthood (Dewey and Begum 2011; Soliman et al. 2021). The lifelong consequences that follow poor nutrition in early life is not only a problem that matters to the quality of life of the victims, rather it is a cross‐cutting agenda impeding economic and societal growth.

Improving breastfeeding and complementary feeding practices is a key determinant for the support of good health that enhances child growth, and reduces child mortality. Limited evidence is available on child feeding among rural communities (Scarpa et al. 2022).

Proper infant and small child feeding practices are crucial for the child's healthy development and growth (Alles et al. 2013). Malnutrition is responsible for preventable morbidity and mortality in babies and young children. The greatest impact has been felt in LMICs, where there has been a major rise in childhood sickness and mortality (Arimond and Ruel 2004; Black et al. 2008; Jannat et al. 2019). Worldwide, more than ten million children under the age of five suffered from starvation, accounting for 60% of deaths (Shabangu 2019). Poor infant feeding practices were responsible for 66% of these deaths in the first 2 years of life (Rathnayake, Madushani, and Silva 2012; Yotebieng et al. 2013).

The World Health Organization (WHO) has developed guidelines for Infant and Young Child Feeding (IYCF) practices for children aged 6–23 months, with Minimum Dietary Diversity (MDD) being one of the core eight indicators (Organization 2021). Globally, only a few children are getting nutritionally sufficient and varied types of foods and in many countries, less than one quarter of infants aged 6–23 months meet the benchmarks for MDD (Belew et al. 2017). Undernutrition is a significant public health problem that prevents children from reaching their full developmental potential. In developing countries, around 32% of children under the age of five are stunted, and 10% are wasted (Organization 2009). In Ethiopia, the prevalence of stunting, wasting, and underweight are 37%, 7%, and 21%, respectively (Indicators 2019).

Mothers' education, wealth quintile, urban residency, home gardening, media exposure, household that produced vegetables and owned animals, a woman who received IYCF education during postnatal care visits, maternal awareness of IYCF, and exposure to IYCF material in the media were all found to be the determinant factors of infants and young children feeding of diversified diet (Agize, Jara, and Dejenu 2017; Edris, Atnafu, and Abota 2018; Getacher et al. 2020). However, there is little data on what are the barriers for infants and young children feeding practices. As a result, this study was aimed to explore the barriers of child feeding practices in rural district, south Ethiopia from primary caregivers and different key individuals' perspectives.

## 2 Methods and Materials

### 2.1 Study Design and Study Area

A qualitative phenomenological study was conducted to explore the barriers to child feeding practices in the rural Gamo zone, southern Ethiopia. This study was conducted in Gamo zone, south Ethiopia. It is one of the administrative zones in the new south Ethiopia region. It bordered the Wolayta, Dawro, and Gofa zones in the North, on the northeast by Lake Abaya, on the southeast by the Amaro Kore zone woreda and Gardulla zone, and on the southwest by South Omo. The administrative center of the Gamo zone is Arba Minch town. Arba Minch town is located 505 km southwest of Addis Ababa, the capital city of Ethiopia. According to the 2007 Ethiopian central statistics agency census, the Gamo zone had a total population of 1,341,901 of whom 668,230 were males and 673,671 were females. Majority of the population, 1,292,653 (96.33%), live in rural areas. Gamo zone has four administrative towns and 15 woreda. It hosted five hospitals (one general and five primary hospitals), 56 health centers, and 299 health posts, which serve the community by providing preventive and curative services. There are a total of 3767 and 587 health professionals and health extension workers (HEWs) in the zone, respectively. This study was conducted in Chencha, Mirab‐Abaya, and Arba Minch Zuria districts from July 14 to September 22, 2023.

### 2.2 Study Participants

The study population included mothers, fathers, HEWs, the Health Development Army (HDA), religious leaders and community elders residing in rural communities of Gamo Zone, south Ethiopia.

### 2.3 Sample Size, and Sampling and Data Collection

Three Focus Group Discussions (FGDs) were conducted, with 8–10 participants per group consisting of mothers and fathers, until data saturation. Additionally, Key Informant Interviews (KIIs) involved three HEWs, three HDA, three community elders, and three religious leaders. All participants were selected using a purposive sampling technique to ensure collection of adequate data. Participants for the FGDs were deliberately selected to gain in‐depth insights and capture a wide range of perspectives and experiences related to the complexities of child nutrition in the local context. The selection criteria included: (1) Caregivers with children aged 6 to 23 months, as their experiences are directly relevant to discussions on child feeding practices; (2) Individuals who are permanent residents of the study area, which enables an understanding of the local culture and traditions that may influence child nutrition and feeding; (3) Gender representation, with three FGDs consisting of fathers and three of mothers, to ensure diverse viewpoints, especially in contexts where gender roles may impact child feeding practices; (4) Couples who are married and living together, as both parents' involvement is crucial for effective child feeding and care, with mutual support potentially affecting feeding practices and access to nutritious food; and (5) Participants who are willing to openly share their experiences and opinions, as their comfort in discussing sensitive topics is essential for collecting honest and valuable data.

Additionally, beyond the aforementioned criteria, individuals involved in local leadership, community volunteer initiatives, and those with direct interactions with caregivers were chosen for KIIs. These individuals can provide insights into the broader community practices and challenges related to child nutrition. As a result, religious leaders, community elders, HEWs, and representatives from women's forums participated in the KIIs to ensure a thorough collection of perspectives and experiences. A pretested semi‐structured interview and FGD guide were used to collect data at the end of the parent study from August 14 to September 10, 2023.

The Focus Group Discussion (FGD) guide was designed to explore the barriers to appropriate child feeding practices in rural southern Ethiopia. It was structured to facilitate a comprehensive discussion about the various barriers to appropriate child feeding practices, emphasizing the importance of understanding the local context. It covers economic, sociodemographic, and sociocultural factors, while also seeking solutions and recommendations from participants. Section one collects basic demographic data from participants, including age, gender, occupation, and educational background. Section 2 aims to explore the economic barriers impacting child feeding practices, focusing on elements like income, employment, and property ownership. It particularly looks into how family income affects the ability to provide nutritious food, prompting individuals to reflect on their own experiences related to income and child nutrition. Moreover, it examines the challenges faced by working mothers in maintaining breastfeeding and ensuring their children receive proper nutrition while balancing their work responsibilities. The section also raises the question of how property ownership affects their capacity to feed their children, linking economic status to child nutrition. Section 3 addresses sociodemographic barriers such as Agro‐Ecological Zone, Mother's Education, Household Head's Education, and Family Size. In this section, the following guiding questions were posed: (1) how does geographical location affect the availability of food and access to nutritious options like fruits and vegetables? (2) What is the relationship between mothers' education levels and their understanding of child nutrition, and what benefits do educated mothers bring to fulfilling their children's needs? (3) How the education of the household head (father or mother) influences child feeding practices? (4) How does the number of children in a household impact nutritional status and feeding challenges? Section 4 of the FGD guide addresses sociocultural barriers that impact child feeding practices, such as polygamy, gender norms, early marriage, freedom of marriage partner choice, feelings of shame or shyness, knowledge of husband's income, support and encouragement, religion, culture, and attitudes. The section poses questions about how these factors influence resource distribution for child feeding, gender roles in feeding practices, the impact of early marriage on nutrition, the influence of partner choice on decision‐making, the effects of societal judgment on feeding choices, the role of husband's income awareness, the importance of familial and community support, the impact of religious beliefs and cultural practices on feeding choices, and the role of caregiver attitudes in feeding practice. The session ends with an overview of the barriers to appropriate child feeding, suggest solutions, and a chance for sharing additional insights.

All data were tape‐recorded, and both FGDs and KIIs were guided by an interview guideline and supplemented by follow‐up and probing questions. The FGDs were moderated by experienced facilitators and assisted by an experienced person from the same culture to facilitate the quality of data collection in the local language. Field notes were also used to supplement the audio‐recorded data. All field data were collected at the nearby health post under optimal conditions. Each FGD lasted from 45 min to 1 h, while the KII lasted between 35 and 45 min. Data collection was terminated after saturation and data adequacy.

### 2.4 Data Quality Assurance

To ensure the quality of the KII and FGD data, various mechanisms were employed, such as recruiting experienced data collectors and facilitators for qualitative studies. Two days of training on conducting KIIs and facilitating FGDs, overseen by senior qualitative research experts, were provided prior to fieldwork. A senior public health expert supervised the entire process daily during the fieldwork. The quality control of the transcripts and translation was conducted on 10% of the audio records by the same senior qualitative research expert.

### 2.5 Data Analysis

Based on the qualitative data presented regarding barriers to appropriate child feeding practices in the rural districts of southern Ethiopia, data analysis was done based on the Social Ecological Model (SEM). The model is a framework that emphasizes the interrelationships between individuals and their environment, considering multiple levels of influence on behavior (Prego‐Meleiro et al. 2020). This framework allows for a more comprehensive understanding of the problem and can inform the development of multilevel interventions to address the identified barriers. It is particularly well‐suited for analyzing the barriers to child feeding practices, as it allows for a comprehensive understanding of the complex factors at play. Therefore, by applying the SEM, the data was analyzed how personal, interpersonal, community, and societal levels interact and contribute to the barriers to appropriate child feeding practices in the rural districts of southern Ethiopia. Voice recorders were used to capture all data, which were then transcribed verbatim in the local language and translated into English by KII/FGD field facilitators daily. Experienced data collectors and supervisors translated the transcripts and back translation was conducted to ensure validity. Supervisors independently verified the transcripts. Thematic content analysis was used to analyze the data, with major themes derived from the study's objectives and subthemes from the text through repeated reading by the research team. Emergent themes were identified and coded by five team members (KF, WGB, MY, and ZHM) using NVivo‐11. The statements were categorized by codes based on related themes. After the themes were determined, the transcripts were reviewed again to confirm that the themes accurately represented the data. All identified themes were validated by the researchers to encompass conversations from the KII/FGDs. The results were presented in narrative form by thematic areas, in line with the study's objective. The quotes featured in the findings are representative of the emerging themes and reflect the typical opinions and views expressed in each KII/FGD.

### 2.6 Ethical Considerations

The study received ethical approval from the Arba Minch University, College of Medicine and Health Sciences Institutional Research Ethics Review Board. Arba Minch University provided a support letter to the respective districts. The recruitment process began with the researchers and assistants introducing themselves to the district chief, explaining the study's purpose, and seeking permission. Before the interviews and FGD, participants were briefed on the study's objectives, invited to take part, and provided with informed consent. KIIs and FGD participants were informed that their involvement was voluntary and that they could withdraw at any time. The privacy and anonymity of the study participants were upheld.

## 3 Results

### 3.1 Characteristics of Participants

A total of 63 individuals participated in the study, comprising six FGDs and 12 KIIs. The mean age of the participants was 34.9 (SD ±6.45) years, with an age range of 22 to 50 years. Slightly over half (58.7%) of the participants were between 30 and 40 years old, followed by the 20–30 age group (28.6%). Half (50.8%) of the participants were female. The majority (55.6%) of participants had completed secondary education or higher. Nearly half (47.6%) of the participants were employed by the government (see Table 1).

**TABLE 1:** Distribution of the sociodemographic characteristics of participants, Gamo zone, south Ethiopia, 2023.

Characteristics	Frequency	Percentage	Remark
Age in years			
20–30	18	28.6	
31–40	37	58.7	
41–50	8	12.7	
Sex			
Female	32	50.8	
Male	31	49.2	
Educational status			
Had no formal education	4	6.3	
Primary	24	38.1	
Secondary plus	35	55.6	
Occupational status			
Farmer	17	27.0	
Government employee	30	47.6	
Merchant	7	11.1	
Private employee	9	14.3	

### 3.2 Barriers to Appropriate Child Feeding Practices

**FIGURE 1:**
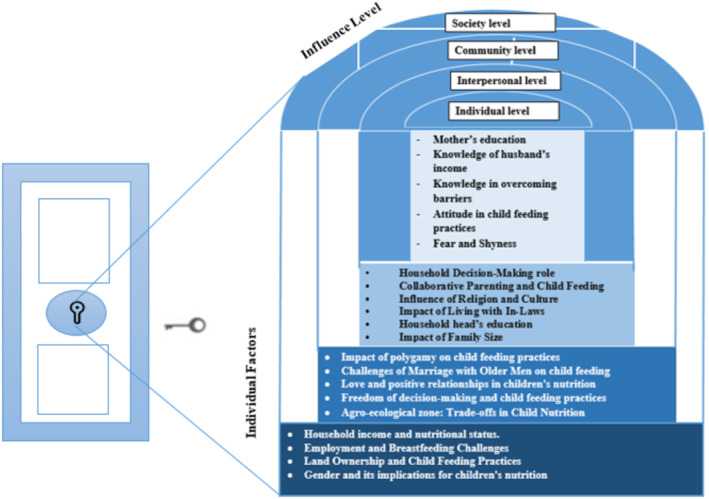
Influence levels of the ecological framework and the factors analyzed in rural south Ethiopia context 2024.

Three primary themes emerged from the FGD and KII data, explaining the barriers to appropriate child feeding practices in the rural Gamo zone. These themes included individual, interpersonal, community, and social levels (see Table 2). The most significant factors involved in the child feeding practice have been classified into the four influence levels (Figure 1). Specific factors were analyzed within each level. Implementation of the new working framework allows the door to be opened, leading to an in‐depth understanding of child feeding practices in rural south Ethiopia.

**TABLE 2:** Summary of the themes and subthemes of barriers to appropriate child‐feeding practices based on the ecological model framework in rural parts of Gamo zone, south Ethiopia, 2023.

Themes	Subthemes
Individual level	Mother's education Knowledge of husband's income Knowledge in overcoming barriers to child‐feeding practices Attitude in child‐feeding practices Fear and shyness
Interpersonal level	Household decision‐making role in child‐feeding practices Collaborative parenting and child‐feeding practices Influence of religion and culture on child nutrition and care Impact of living with in‐laws on child nutrition Household head's education Impact of family size on child nutrition
Community level	Impact of polygamy on child‐feeding practices Challenges of marriage with older men on child feeding Love and positive relationships in children's nutrition Freedom of decision‐making and child‐feeding practices Agro‐ecological zone: Trade‐offs in child nutrition
Societal level	Household income and nutritional status Employment and breastfeeding challenges Land ownership and child‐feeding practices Gender and its implications for children's nutrition

### 3.3 Theme 1: Individual Level

#### 3.3.1 Subtheme 1.1: Mother's Education

The study participants emphasize the crucial role of education in shaping child feeding practices. Educated women are proactive and aware of nutritional needs and breastfeeding guidelines, unlike uneducated women who may lack awareness of proper feeding practices. The quote highlights the knowledge gap among uneducated women, which can lead to inadequate nutrition for their children despite having sufficient resources. Even when uneducated women have access to food supplies, their lack of knowledge prevents them from preparing a balanced diet. This underscores the importance of education in enabling mothers to make informed decisions about their children's nutrition.

There is a difference between educated and uneducated women. Educated women plan for the needs of their newborns, including the duration of breastfeeding and the appropriate diet for the child. On the other hand, uneducated women tend to take the fact of being alive as a blessing, and may not be aware of the benefits of following recommended feeding practices. Even when they have enough household supplies, they may not be able to prepare a balanced diet for their child due to a lack of knowledge. [43 years old male FG discussant]

#### 3.3.2 Subtheme 1.2: Knowledge of Husband's Income

The study participants emphasize how a lack of transparency in household income can hinder effective child feeding practices. Women who are unaware of their husband's income may make unrealistic plans for their children's nutrition, leading to conflicts within the family. On the other hand, when women have knowledge of the household's financial situation, they can make informed decisions and create affordable nutrition plans for their children. This highlights the importance of financial literacy in effectively allocating resources for children's nutritional needs.

If I do not know his income, I may plan more than he can afford. When the household income is known plans are made as per the income level. What I saw from the neighbors, what I learned from healthcare workers make me ask more and more above the income level. Therefore, knowing the income level makes women prepare affordable plans. This difference brings argument, crisis, and mistrust. [37 years old male, FG discussant]

Not only knowing the income of the husband but also common understanding on the income and opening the common bank account for saving is important.

Saving money together will not be affected. If they consult and open a bank book together and save together, they have discussed what will happen to the house and avoided what will happen, but they may not save it completely. Therefore, it does not affect children's nutrition. [43 years old female health extension worker, KII]

#### 3.3.3 Subtheme 1.3: Knowledge in Overcoming Barriers to Child Feeding Practices

The study participants in this research emphasize the importance of knowledge and understanding in overcoming barriers to child feeding practices, even when resources are available. Despite having access to nutritious foods like milk, poultry, vegetables, and fruits, some families prioritize selling these products in the market rather than using them to provide a balanced diet for their children. The statement underscores the significance of proper knowledge and understanding in utilizing resources effectively to ensure children receive adequate nutrition. Keeping small livestock, growing vegetables and fruits, and having dairy products can contribute to a balanced diet for children. However, a lack of knowledge often leads to neglecting children's feeding needs. The lack of knowledge and understanding hinders families from providing optimal nutrition for their children, even when resources are available.

…they say, “children grow up by their luck.” Even having income and milk in their house, they sell it in the market. Some poultry products available in the house, vegetable and cereals, meat products. When children are sick the products they hinder and sell in the market will not pay the medical fee ….it is easy to get good feeding, small poultry farming in the house, beekeeping, vegetables, fruits like mango, and dairy from one or two cows is enough to feed them a balanced diet. Due to lack of understanding or knowledge people ignore children feeding. [37 years old merchantman, FG discussant]

#### 3.3.4 Subtheme 1.4: Attitude

The study participants highlight the importance of attitude in child feeding practices, emphasizing that wealth does not guarantee good nutrition for children. Caregivers' attitudes play a crucial role in ensuring children's health and nutrition, regardless of financial status. Individuals from wealthier backgrounds may neglect their children's nutritional needs due to a lack of concern, while those with fewer resources may be more dedicated to their children's health. This underscores the significance of a proactive and caring attitude towards child feeding, which can be a barrier when absent.

… some might be rich but not care about their child's health and feeding, on the contrary, the poor might care more and do whatever is expected from them. So, in my opinion the most important thing is attitude not money. [30 years old female health extension worker, KII]

#### 3.3.5 Subtheme 1.5: Fear and Shyness

The study participants emphasize how a young wife's fear and shyness towards her older husband can create barriers to ensuring adequate nutrition for her children. The wife may feel constrained in expressing her needs or those of her children due to a sense of moral obligation to her husband and a fear of being insulted or disrespected. This lack of freedom to communicate openly with her husband can result in the wife being unable to ask for essential items like food or money for her children. This fear and shyness can directly impact the children's well‐being by hindering their nutritional needs from being met.

Young wives are afraid to ask their old husbands for something. There is a fear spirit. To keep the moral of her old husband. He is my father's friend. How can I ask him, may he insult me? This fear and lack of freedom may impose an effect on her children…fear to ask for consumable items, fear to ask for food items, fear to ask money to buy things from the market and the like. [40 years old farmer man, FG discussant]

### 3.4 Theme 2: Interpersonal Level

#### 3.4.1 Subtheme 2.1: Household Decision‐Making Role in Child Feeding Practices

Key informants emphasize the role of household decision‐making in food and nutrition. The husband is primarily responsible for financial matters, while the mother determines food needs for the household and children. However, this division of roles can hinder effective child feeding practices. Challenges may arise if the husband controls finances, limiting the mother's ability to access necessary resources for child nutrition. Additionally, the mother's knowledge may not translate into action if the husband makes financial decisions. This dynamic could lead to conflicts and inadequate funds for children's dietary needs, impacting their health and development.

…It is the husband's responsibility to spend money, but since it is the wife who knows everything that is needed for household consumption, everything that is bought for the house is bought through her. It is the mother who is the one who buys the things that are needed for the children. [35 years old male community elder, KII]

#### 3.4.2 Subtheme 2.2: Collaborative Parenting and Child Feeding Practices

The importance of parents working together for the positive growth and development of children is highlighted in the discussion. Lack of collaboration and cultural expectations can act as barriers to effective child feeding practices. If one parent is not involved, it may lead to imbalances and deficiencies in nutrition. The need for shared responsibilities and mutual support between partners is emphasized to ensure nutritious meals for children. Understanding these dynamics can help in developing interventions to improve child feeding practices and overall health.

…it will be good for the child and it will make the children grow up well. It makes wife and husband common and then they raise children together as long as they are common. [37 years‐old male community elder, KII]

#### 3.4.3 Subtheme 2.3: Influence of Religion and Culture on Child Nutrition and Care

Key informants in the community highlight a prevalent cultural belief that sees malnutrition as a consequence of divine punishment rather than nutritional deficiencies or lack of care. This belief results in families not seeking medical aid for malnourished children, perpetuating cycles of malnutrition and poor health outcomes. This leads to delayed treatment, continued malnutrition, and lack of preventive care, all of which can have long‐term negative effects on children's health and well‐being.

… we find malnourished children, the community will not be willing to take the child to the health facility. They think the problem is related to God's punishment and not malnourishment. Sometimes we might even ask for help from the police when the family is reluctant. This is due to the influence of the culture in this area. [30 years‐old female health extension worker, KII]

#### 3.4.4 Subtheme 2.4: Impact of Living With In‐Laws or a Third Party on Child Nutrition

The participant suggests that living with in‐laws or third parties can lead to neglect of children's basic needs, including proper nutrition. Caregivers may not inquire about hunger or thirst, leading to inadequate feeding practices. Children may feel fearful and insecure, inhibiting their ability to communicate their needs. Mothers may face limited autonomy in advocating for their children's needs, impacting feeding and care. Cultural dynamics may prioritize different values that do not align with modern nutritional needs, perpetuating neglect and poor health outcomes for children. Interventions are needed to support mothers in advocating for their children's nutrition in rural contexts where living arrangements can impact feeding practices and child health. Understanding these barriers is crucial for addressing child nutrition challenges.

I think living with a third party is very harmful. Anyone will not see your child as a child because they think that they are only human and may not ask if they are hungry or thirsty. The children are afraid. [39 years old female community elder, KII]

#### 3.4.5 Subtheme 2.5: Household Head's Education

The research highlights the crucial influence of community figures, such as knowledgeable fathers and elders, in determining child feeding practices and the overall nutritional status of children. Fathers with education are more inclined to make well‐informed choices regarding suitable feeding behaviors, resulting in improved nutritional outcomes for their offspring. Additionally, they foster a supportive emotional atmosphere during mealtimes, which promotes healthy eating habits among children. Conversely, fathers who lack education may inadvertently perpetuate detrimental feeding practices, thereby increasing the responsibilities placed on mothers. Consequently, there is a pressing need for educational initiatives aimed at fathers to enhance child nutrition. By bridging the educational divide among fathers, it is feasible to refine child feeding practices and ultimately elevate the overall nutritional well‐being of children in rural areas.

If the father is educated, it will help him to take care of his children and identify child feeding behaviors and attempts to adhere to it. Unless, there can be a possibility to cause undue pressure during a meal. You know, we men sometimes become inpatient while feeding because we want everything to be easy going, but the child may not be. It is the skill or awareness that helps you through ……… [37 years old male religious leader, KII]

#### 3.4.6 Subtheme 2.6: Impact of Family Size on Child Nutrition

Study participants suggest that larger family sizes can impact child nutrition negatively in multiple ways. Firstly, resource allocation may be stretched thin, resulting in inadequate nutrition for each child due to limited food resources. Secondly, as family size increases, the demand for nutrition also rises, potentially leading to disparities in nutritional status if families struggle to provide sufficient and nutritious food. Additionally, larger families may mean that parents have less time and attention to focus on each child during mealtimes, affecting feeding practices and overall eating environments. This can lead to children not being encouraged to eat properly. The burden of managing a larger family can also lead to stress for mothers, affecting their ability to ensure all children receive adequate nutrition. Understanding these barriers is crucial for developing interventions to improve child nutrition in families with multiple children.

If the family size is large, the nutritional status will decrease. The nutritional status of a child of one or two is different from that of a child of five or six. [35 years old male community elder, KII]

### 3.5 Theme 3: Community Level Factors

#### 3.5.1 Subtheme 3.1: Impact of Polygamy on Child Feeding Practices

The participants in the study examine how polygamy influences child feeding practices, drawing attention to disparities in care and financial limitations. The presence of multiple spouses results in unequal attention given to children, which adversely affects effective feeding methods. Financial constraints further complicate feeding situations in polygamous households, especially in rural regions where resources are limited. This highlights the systemic nature of the problem, placing it within the broader community framework. By concentrating on practical consequences, the discussion reveals the distinct obstacles encountered by families in polygamous environments, extending beyond emotional factors and the distribution of attention, and directly impacting the nutrition and health of children.

Polygamy is prevalent within our community, and it has a significant impact on the feeding practices of children. Specifically, those who have children from two or three wives experience unequal care for their offspring. This discrepancy arises when there are multiple children to support but limited income to meet their needs. [35 years old man FG discussant]

#### 3.5.2 Subtheme 3.2: Challenges of Marriage With Older Men on Child Feeding

The participant highlights when an aged man marries a young woman, the childcare and feeding duties fall primarily on the young wife. The husband's advanced age makes it difficult for him to actively participate in feeding the child. The young wife's lack of knowledge about proper infant feeding practices, due to her immaturity, can negatively impact the child's growth and development.

If the age difference is that a very old man marries a too young girl, there will be challenges in her life even. When we have children, the aged man marries young women, then the care, feeding, etc. duties are on the shoulders of the women. Therefore, children's growth and development is problematic. [38 years old daily laborer man FG discussant]

In some cases, the young wife's disdain for her much older husband and lack of communication can prevent her from learning important skills like preparing appropriate food for their child.

Older husbands do not accept the ideas of young women. Misunderstanding is there, ignorance from the younger women's side. You are too old, equal to my father. She disdains his husband, then there will be a lack of communication, and learning. In this circumstance she may not prepare appropriate food for the child. [37 years old employed man, FG discussant]

In addition, the young wife's lack of awareness about healthy nutrition for children can hurt the infant. She may not understand what foods to buy and provide to her child. In addition, marrying at an immature age means the wife will have a limited understanding of infant nutritional needs. This can have a significant impact on the child's health and development. It is recommended by science that couples marry when they are mature enough. Marrying at a mature age allows the wife to have a better awareness of healthy nutrition for infants and children and provides the wife with a better understanding of infant feeding practices and nutritional needs. This allows her to make more informed decisions about what to feed her child.

As it is recommended by science, if they get married when they are mature enough, they will have a better mind. Better awareness will also have a better understanding of healthy nutrition for children. But if this is not the case, it will hurt them. It means that they will not understand what to buy for children. It makes an impact. [36 years old male religious leader, KII]

#### 3.5.3 Subtheme 3.3: Love and Positive Relationships in Children's Nutrition

The study participants suggest that the dynamics of marital relationships, particularly the emotional aspects, play a significant role in influencing children's nutrition and overall well‐being. The participant also emphasizes the importance of voluntary and loving relationships in marriage. It suggests that when couples marry out of love and mutual choice, it fosters a nurturing environment that positively impacts children's nutrition.

In addition, the assertion that “if he loves her, he will love his son too” implies that a father's affection for the mother is linked to his care for the child. This reflects the idea that positive emotional bonds within the family can lead to better parenting practices and, consequently, better nutritional outcomes for children.

Conversely, the statement acknowledges that if a husband harbors negative feelings towards his wife, it may extend to his feelings towards their children. This suggests that conflict or lack of affection in the marital relationship can lead to neglect or inadequate care for children, adversely affecting their nutritional status.

If they get married willingly and by choice, there will be no impact on children's nutrition. The main thing is love! If he loves her, he will love his son too, no problem. If some husbands hate her, some will hate their children too. [43 years old female health extension worker, KII]

#### 3.5.4 Subtheme 3.4: Freedom of Decision‐Making and Child Feeding Practices

The participants discussed the implications of marriages that are formed out of societal pressure rather than mutual consent. It highlights how such unions may lack the freedom of decision‐making, particularly regarding child feeding practices. The pressure to marry due to an unplanned pregnancy can lead to relationships that do not prioritize the best interests of the child. This is relevant to the study objective as it illustrates how societal norms and lack of personal choice in marriage can create barriers to effective child feeding practices, as parents may not work collaboratively or prioritize their children's nutritional needs.

Marriage based on unwillingness of the two partners is not sustainable. In our community if pregnancies happen before marriage, people try to let them marry each other. This is to escape punishment and societal stigma from the women's side. This kind of marriage will not come to freedom of decision on child feeding. [48 years old farmer FG discussant]

#### 3.5.5 Subtheme 3.5: Agro‐Ecological Zone: Trade‐Offs in Child Nutrition

The study participants in the highland agro‐ecological zone prioritize selling dairy products over using them to feed their children, believing that the financial gain will benefit their families. This decision creates a trade‐off between income and child nutrition, with mothers sacrificing their children's nutritional needs for immediate financial resources. The tension between economic necessity and child feeding practices highlights the challenges faced by mothers in making decisions about nutrition in this specific context.

In the highlands, most mothers decide against feeding their kids eggs and instead bring them home to sell. Because milk is costly, it is sold. They realize they took the money. They make butter, which they take to market. They churn the butter and sell it in the market. But if they sell at least 75 percent, 25 percent can take care of their children at home. [25 years old female HDA, KII]

### 3.6 Level 4: Societal Level

#### 3.6.1 Subtheme 4.1: Household Income and Nutritional Status

This subtheme succinctly captures the fundamental link between household income and nutritional status. The remarks made by participants clearly associate low income with insufficient nutrition and adverse health outcomes, which is crucial for comprehending the effects of household income on the well‐being of children. Furthermore, it highlights the wider implications for children's health, offering a comprehensive understanding of the matter. The speaker, recognized as a male religious leader, lends an authoritative voice and a community‐oriented perspective to the discussion, indicating that this viewpoint is likely to resonate with others within the community.

…If the family has a low income, they will not eat properly. Without proper nutrition, good health is not possible. Therefore, the economic status of the family is closely linked to the health and nutrition of the children. [36 years old male religious leader, KII]

#### 3.6.2 Subtheme 4.2: Employment and Breastfeeding Challenges

Discussant directly addresses how maternal employment can negatively impact exclusive breastfeeding practices: highlighting the challenges faced by employed mothers in continuing to breastfeed their children after returning to work. When mothers have to rely on others for childcare, it can disrupt the breastfeeding process and deprive the child of the benefits of breastmilk.

Employed women often have to return to work after giving birth, leading them to entrust the care of their child to a family member or maid. This can sometimes result in the child being unable to breastfeed, missing out on the benefits of breastfeeding. Despite this, the mother may still be able to prepare homemade remedies for her baby while at work. [50 years‐old male FG discussant]

#### 3.6.3 Subtheme 4.3: Land Ownership and Child Feeding Practices

Participant suggests that in the community, having a large plot of land is essential for growing food to feed one's children. However, most people in the area face a shortage of land, and even those who own land often fail to cultivate it. This lack of land availability and underutilization of existing land will inevitably have negative consequences for the community's ability to provide adequate nutrition for children. In addition, it emphasizes the importance of land ownership as a means of ensuring proper child feeding practices. It also highlights the challenges faced by community members who lack access to sufficient land for cultivation, which can lead to food insecurity and potential malnutrition among children.

…In our area, having a large plot of land is necessary to sow and provide adequate food for one's children. Unfortunately, most people in our community have a shortage of land. While some individuals do own land, they fail to cultivate it. This issue of limited land availability will inevitably have an impact on our community. [36 years‐old male religious leader, KII]

#### 3.6.4 Subtheme 4.4: Gender and Its Implications for Children's Nutrition

The participants in the study recognized several obstacles to effective child feeding practices and nutrition in rural settings. Among these obstacles is the gendered division of labor, which assigns caregiving responsibilities primarily to women, thereby limiting men's participation in child care and nutritional matters. Financial constraints and the control of resources by men further restrict women's capacity to procure sufficient food, resulting in conflicts and misunderstandings about household requirements. This power imbalance influences decision‐making processes, as women's choices are frequently challenged or diminished, adversely affecting the quality of food accessible to children. Moreover, such dynamics can foster a stressful atmosphere for women, which in turn affects their ability to care for children effectively. These barriers illustrate systemic challenges within rural communities that impede child nutrition, underscoring the necessity of addressing gender dynamics to enhance feeding practices and overall child health in these regions.

Men think washing and feeding is her task. When giving money to buy food items men limit the amount, then she says this is not enough, he says how not enough? A few days later if she asks him again, he will say the last day you bought a lot, where did it go? This stuff of argument and dialogue is not appropriate for her. This hampers her decision‐making capacity and freedom. [40 years‐old farmer man, FG discussant]

## 4 Discussion

In Ethiopia, efforts are being made to implement a multi‐sectoral plan of nutrition intervention (as prescribed in the Sekota Declaration and National Nutrition Program) to end the high burden of undernutrition by 2030 (Banerjee 2013). However, the country is still experiencing one of the worst scenarios in child feeding practices due to different barriers (Geda et al. 2021). This study focuses on exploring the Barriers to Optimal Child‐Feeding Practices in Rural Gamo Zone, South Ethiopia: A Qualitative Exploration of Caregivers' and Key Stakeholders' Perspectives.

According to the thematic analysis of this study, economic status plays a significant role in determining the appropriate child‐feeding practices that parents or caregivers can adopt. It was explored that economic status impacts the types and quality of food available within a household. Families with higher economic status often have better access to a variety of nutritious foods, such as fruits, vegetables, meats, and dairy products. These resources enable parents to provide well‐balanced meals for their children, ensuring they receive the necessary nutrients for healthy growth and development. Conversely, families with limited financial resources may depend more on inexpensive, but potentially less nutritious, options like processed foods, or cheaper staple foods. This finding is consistent with previous qualitative studies conducted in north‐west Ethiopia (Gizaw, Sopory, and Sudhakar 2023), south‐western Uganda (Scarpa et al. 2022), the Democratic Republic of Congo (Burns et al. 2016), rural Rwanda (Ahishakiye et al. 2019), and east Nigeria (Onah et al. 2014).

Household head the economic power entailed to him affects the power over decision‐making for the expenditures in the household. This influenced what foods were prepared, who eats different kinds of foods, and how money was spent. Study in Ghana revealed that a child's father having adequate income were important determinants of the consumption of an adequate diet by the children. Our finding was supported by previous research where house head affects purchase of food items which affect health and nutrition practices (Bimpong et al. 2020; Burns et al. 2016). This was also confirmed by another study that identified that one of the barriers to optimal child feeding identified were lack of money to purchase the nutritious foods recommended for children (Armar‐Klemesu et al. 2018).

This study also explored that sociocultural aspects play a crucial role in shaping appropriate child feeding practices. In societies where polygamy is common, the presence of multiple wives and their respective children can create competition for resources, including food. This can lead to inequality in child feeding practices and potentially affect the nutritional status of children in polygamous households. In communities where early marriage is prevalent, adolescent mothers may lack the necessary knowledge and support to provide adequate nutrition to their children. Cultural norms around modesty and shame can sometimes hinder women from seeking information and support related to child feeding practices. In households where men control the finances, women may have limited resources to ensure adequate nutrition for their children. This conclusion aligns with previous qualitative research conducted in north‐west Ethiopia (Mekonnen et al. 2018), western Ethiopia (Assefa et al. 2021), the Democratic Republic of Congo (Burns et al. 2016), and rural Ghana (Armar‐Klemesu et al. 2018).

One of the barriers for child feeding practices arises from the culture and religious view of the communities. Some of the cultures in developing countries sometimes give much emphasis for the male and forgets women and young children. This study reported a similar finding in that gender imbalance in the culture is affecting the nutrition practices of the young children. It affects resource distribution that may affect food or feeding in the household. This type of cultural practice affects the feeding and nutrition of the young children in the household. Similar findings were reported from study of gendered social and cultural norms that influence intra household relationships and are also known to influence child health and nutrition outcomes (Mwaseba and Kaarhus 2015; Seebens 2011). However, it is in contradiction with a study conducted on the role of women empowerment that reported children of empowered women belonging to male‐headed households had poorer nutritional status than those in households with empowered female heads. The study shows that women's empowerment, particularly in asset ownership and decision‐making autonomy, is protective against childhood nutritional anemia (Christian et al. 2023).

An unfavorable attitude towards child feeding can have a barrier for appropriate child feeding practices. When parents or caregivers have a negative view of feeding, they may unknowingly adopt ineffective or harmful practices that can compromise the child's nutritional intake and overall health. Parents with an unfavorable attitude may restrict certain food groups or put strict limits on portion sizes, fearing that the child will become overweight or develop unhealthy eating habits. However, this approach can lead to nutrient deficiencies and an imbalanced diet, as well as potentially contributing to disordered eating behaviors later in life. On the other hand, parents may have an unfavorable attitude towards a child's low appetite or picky eating behaviors. This can lead to excessive pressure to eat, which can cause stress and anxiety around mealtimes for the child. The findings of this study are in line with earlier quantitative research carried out in Ghana (Bégin and Aguayo 2017), South Ethiopia (Bégin and Aguayo 2017), and Northern India (Nikhurpa, Nikhurpa, and Pangty 2021).

Agro‐ecological zones like low land highlands can affect the availability and diversity of food. Even though they do not inherently hinder appropriate child feeding practices, they have an effect on food production that can affect the feeding practices and also nutrition of the infants and children. In regions with limited access to fresh fruits and vegetables due to unfavorable agriculture to provide an optimal diet for children. In this study among commonly mentioned themes was the effect of highland verse lowland which are agro‐ecological zones that may pose challenges in terms of food availability or nutrient‐rich options, which can indirectly influence child‐feeding practices. Food production and availability of the food for purchase are among the barriers that affect the feeding practices of the children. One of the qualitative studies from Ghana reported that seasonal food insecurity is one of the barriers for appropriate child feeding practices (Armar‐Klemesu et al. 2018) and also another review confirmed that food insecurity is one of the barriers for appropriate practice of child feeding (Bazzano et al. 2017).

Education is one of many factors that contribute to appropriate child feeding practices in many settings. Evidence suggests that the education of the father can influence the mother's knowledge and attitude which will contribute to the better nutrition of the child. In this study, education of the father was one of the key factors rising as affecting the feeding practice of children. Similar reports were reported from South west Ethiopia when father's educational status positively affects the feeding and nutrition of children (Onah et al. 2014).

Maternal educational status is one of the barriers for practicing appropriate child feeding. Education gives the knowledge and attitudes to practice appropriate child feeding. Our finding is in agreement with a study conducted in Nigeria and Ethiopia where low maternal education affects the child feeding practice (Assefa et al. 2021; Onah et al. 2014). Education is one of the tools for information gathering. Educated person can access information from different sources. One of the studies conducted in Uganda found that lack of breastfeeding and complementary feeding information has affected the breastfeeding and complementary feeding practices of children (Scarpa et al. 2022).

Maternal education has a big impact on child nutritional status. It is commonly discussed by discussants as major barriers for appropriate child feeding practices. A study found that children with less educated mothers were significantly more likely to be stunted. Maternal education also affects the prevalence of food insecurity among households which is basically the determinant for the feeding practices of children. Similar study reported that households with food insecurity and less educated mothers were more likely to have malnourished children (Ajao et al. 2010).

One of the key barriers that affect the nutrition and feeding practices commonly reported were lack of nutrition knowledge and experience, receiving conflicting messages from different sources (Athavale et al. 2020). In this study, it was stated that education may affect the need and practice of child feeding. Good and appropriate knowledge regarding the needs of the children will affect deciding what is needed for them. Different studies report that maternal knowledge affects child nutrition and growth (Iftikhar et al. 2017). In other ways mothers' knowledge, attitudes, and practices of breastfeeding are affected by the mother's well‐being (Witten et al. 2020).

Another barrier for the appropriate practice of child feeding was food safety knowledge and practice (Sandow et al. 2022). Similar study conducted in Ghana on mothers who have children 6–23 months regarding their knowledge and attitudes of child feeding recommendations, complementary feeding practices, and determinants of adequate diet identified that mother's knowledge regarding IYCF recommendations (Bimpong et al. 2020).

Another barrier for better child feeding practices is caregiver beliefs, practices, and nutrition knowledge gaps. This theme was among commonly emerging themes during FGD and KIIs. The beliefs that parents have affects infant and child feeding practices. A study from Ghana reported a similar finding that caregiver's beliefs affect the nutrition practices of the children. Education helps to bridge the gap between knowledge and practice. Proactive nature of the household or the caregiver includes recognition of the dietary needs of young children and commitment to provide foods to meet these needs (Armar‐Klemesu et al. 2018).

Support from family members and husbands also affects the feeding practices of the children. A study from south west Ethiopia identified that father's involvement and support (Armar‐Klemesu et al. 2018). Another study from Ghana identified that support from family, friends, and community members are among the facilitators for the practice‐appropriate child feeding (Sandow et al. 2022).

Parental encouragement to eat practice is a positive, gentle, supportive, and non‐coercive practice to build on a child's healthy eating behaviors, and allow children‐appropriate feeding practice. Caregiver feeding practices of infants and young children present one important set of modifiable behaviors among mothers. Mothers are the key actors for child feeding practices (Ickes et al. 2018). One qualitative review identified that lack of support and encouragement affect the breastfeeding practice, which in turn affects overall feeding practices of children (Bazzano et al. 2017). Strengthening social support for mothers and expanding overall maternal capabilities hold potential for addressing important underlying determinants of child feeding is recommended as one of the strategies for improving child feeding practices (Ickes et al. 2018).

Women's empowerment has gained attention as critical for child nutrition during the first 1000 days of life. Support for a woman from a family or others is one of the empowerments for the women. This has an impact on child nutrition. Our finding was supported by a study from India that reported that limited social support for maternal decision‐making affects child feeding practices (Athavale et al. 2020). This women's empowerment, the expansion in women's ability to make strategic life choices usually affect the decision that will affect the well‐being of the children (Kabeer 1999). Therefore, it is agreed that empowerment and family support are among the key facilitators for improved child feeding practices (Athavale et al. 2020).

Similarly, social support was commonly mentioned as one of the facilitators or barriers for appropriate child feeding practices. This was because provision of social support along with improved information and education of influencers (fathers/father‐in‐law and grandmothers or mother‐in‐law) can impact IYCF practices both indirectly by supporting mothers and directly by the family members implementing optimal feeding practices themselves (Bazzano et al. 2017). Studies from Kenya showed that engaging fathers and grandmothers of infants to improve their knowledge of optimal infant feeding practices and to encourage provision of social support to mothers could help improve some feeding practices (Adhisivam et al. 2017; Mukuria et al. 2016). Similarly, another study from Rwanda reported that the involvement of the father in child feeding positively affects consumption of animal source food to improve child feeding practices (Flax et al. 2023).

Family size is one of the barriers that affect the feeding practices of the children. As the number of families increases it creates another pressure on the family resources which affect the decision to share the resources among the family members. This will negatively affect child nutrition. The problem will be exacerbated if the family is poor. This can stress family in general and women in particular. Women are usually stressed when they fail to provide the necessary food items for child feeding and nutrition. Many studies have documented the strong impact of family size and education on growth parameters of their children (Ajao et al. 2010). Susan has demonstrated family size carries a substantial effect on children's growth which is the result of good nutrition practice (Horton 1986). A contemporary study done in Bangladesh has supported the strong association of maternal education, working mothers, and family size on child's well‐being and growth (Ahsan et al. 2017).

## 5 Conclusions

The present study highlighted that limited income and employment opportunities, absence of property ownership, restricted education for mothers and husbands, a high number of children, polygamous households, gender disparities, early marriages, limited freedom in choosing a partner, shyness, lack of awareness about the husband's income, lack of support from family or community, and cultural and religious beliefs were barriers to child feeding in rural areas.

Having a large family and polygamous households' resources may be divided among multiple families, potentially compromising the ability to ensure adequate nutrition for children. In gender inequalities, girls often receive less food and nutrition compared to boys. Early marriages can disrupt the education of adolescent girls and impact their ability to provide appropriate child feeding practices due to limited knowledge and resources.

Furthermore, shyness or cultural norms surrounding discussions about sensitive topics like child feeding practices can hinder access to information that would promote appropriate feeding. Lack of awareness about the husband's income can create uncertainty in budgeting for adequate child feeding practices. Lack of support from family members or the community can further impede parents' ability to provide appropriate child feeding practices. Cultural and religious beliefs may also influence child feeding practices, potentially limiting access to certain types of food or imposing restrictions that affect the nutritional quality of meals.

A multi‐sectoral strategy comprising education, economic empowerment, and supportive legislation should be developed and widely available to enable optimal child feeding practices. We can create an environment in which all children, regardless of socioeconomic background or cultural context, have access to the nutritious food and care they require to develop and flourish by implementing multiple interventions.

## Author Contributions

K.F. conceptualized and designed the study, participated in patient recruitment, data analysis, and data interpretation, drafted the initial manuscript, and was responsible for the overall content as the guarantor. M.Y. and W.G.B. participated in data analysis, and data interpretation, and drafted the first manuscript. Z.H. participated in study design, and data analysis, interpretation, and preparation of the first manuscript. All the authors have critically reviewed it for significant intellectual content; have approved the final manuscript as submitted.

## Conflicts of Interest

The authors declare no conflicts of interest.

## Data Availability

The data used in this report is available on qualitative data repository.
